# Aryl Hydrocarbon Receptor Mechanisms Affecting Chronic Kidney Disease

**DOI:** 10.3389/fphar.2022.782199

**Published:** 2022-02-14

**Authors:** Colleen S. Curran, Jeffrey B. Kopp

**Affiliations:** ^1^ Critical Care Medicine Department, Clinical Center, NIH, Bethesda, MD, United States; ^2^ Kidney Disease Section, NIDDK, NIH, Bethesda, MD, United States

**Keywords:** RAAS, aryl hydrocarbon (Ah) receptor, kynurenine, hypoxia, PPAR γ, TGF—β1

## Abstract

The aryl hydrocarbon receptor (AHR) is a basic helix-loop-helix transcription factor that binds diverse endogenous and xenobiotic ligands, which regulate AHR stability, transcriptional activity, and cell signaling. AHR activity is strongly implicated throughout the course of chronic kidney disease (CKD). Many diverse organic molecules bind and activate AHR and these ligands are reported to either promote glomerular and tubular damage or protect against kidney injury. AHR crosstalk with estrogen, peroxisome proliferator-activated receptor-γ, and NF-κB pathways may contribute to the diversity of AHR responses during the various forms and stages of CKD. The roles of AHR in kidney fibrosis, metabolism and the renin angiotensin system are described to offer insight into CKD pathogenesis and therapies.

## Introduction

Chronic kidney disease (CKD) affects approximately 37 million (1 in 7) US adults and is the ninth leading cause of death (CDC, 2020). The global incidence of CKD has been estimated at 13.7% (11.7–15.1%) of the nearly eight billion people, or over one billion cases. Current therapies may slow and occasionally halt CKD progression, but most patients continue to progress. Further, these therapies are not fully available to or fully used by many of the globally-affected individuals. CKD is accompanied by premature morbidity and mortality from cardiovascular disease, as hypertension and uremia combine to drive cardiac and vascular damage.

CKD can be caused by systemic diseases and by primary kidney diseases. CKD due to systemic disease is more common; causes include diabetes mellitus (Navaneethan et al., 2021), malignant hypertension (van den Born et al., 2005), systemic lupus erythematosus and systemic vasculitis (Moiseev et al., 2020). Primary kidney diseases, those that chiefly affect the kidney, include polycystic kidney disease, interstitial nephritis, and podocytopathies (minimal change disease, focal segmental glomerulosclerosis and membranous nephropathy) (Curran and Kopp, 2021).

Many CKD patients progress to end-stage kidney disease. Kidney transplant extends life for chronic dialysis patient by a median of 12 years (2014–2017: median 11.7 for deceased donor kidney recipients, median 12.1 years for living donor kidney recipients) (Poggio et al., 2020) and longer in some cases (Zolota et al., 2020). Many patients on chronic dialysis, particularly in the First World, are older and have co-morbidities; their 5-year survival rate has been reported as 56% (Nordio et al., 2012).

Increased rates of cardiovascular disease in CKD patients are the major driver of reduced survival (Jankowski et al., 2021). Thus, better strategies are needed to prevent CKD and to slow or halt progressive loss of kidney function in individuals with CKD.

CKD incidence is linked to several risk factors. Non-modifiable risk factors include genetic variants, low birth weight, and older age. Notable genetic variants include polymorphisms in angiotensin-I converting enzyme (*ACE*), angiotensin II type 1 receptor (*AGTR1*) and apolipoprotein L1 (*APOL1*), the latter accounting for much of the excess CKD risk among populations with sub-Saharan ancestry. Common modifiable risk factors include smoking, obesity, hypertension, diabetes mellitus, excessive alcohol consumption, heavy metal exposure, and excessive use of analgesic medicines (Kazancioglu, 2013). These factors compromise or alter kidney cell function, affecting cells in the glomerulus (podocytes, mesangial cells, and endothelial cells) and in the tubulo-interstitium (tubular cells, endothelial cells, and fibroblasts).

The various factors that induce CKD allow for the development of animal models to mimic the response. These can include 5/6 nephrectomy, unilateral ureteral obstruction nephropathy, angiotensin II (Ang II)-induced hypertension, ischemia/reperfusion-induced acute kidney injury, cisplatin- or cyclosporin A-induced nephropathy or models of systemic disease. An underlying factor in animal or human CKD progression is the aryl hydrocarbon receptor (AHR). This nuclear receptor is activated by endogenous and exogenous ligands and is required for normal kidney development and function. In cultures of murine metanephros, the AHR ligand, benzo(a)pyrene (BaP), disrupted nephrogenesis (Falahatpisheh and Ramos, 2003) and in fetuses from mice fed the AHR ligand 2,3,7,8-tetrachlorodibenzo-p-dioxin (TCDD, dioxin), hydronephrosis was a common malformation (Peters et al., 1999). AHR renal mRNA levels were also higher in female rats compared to males and at 7 weeks post-5/6 nephrectomy, AHR renal mRNA levels decreased in females, but not males, highlighting a role for AHR in renal gender differences (Lu et al., 2006).

In an animal model of CKD, AHR is not only activated in the kidney but additional organs. Specifically, transgenic mice expressing the AHR responsive-promoter tethered to a β-galactosidase reporter gene and subjected to ischemia/reperfusion-induced acute kidney injury identified AHR activation in the proximal and distal renal tubules, cardiac myocytes, hepatocytes, and microvasculature in the cerebral cortex (Walker et al., 2020). This suggests a presence of AHR ligands in serum, which can activate cells in the kidney, heart, brain, and liver. By culturing serum with an AHR reporter cell line, elevated AHR activity was identified in diabetic nephropathy patients compared to controls (Kim et al., 2013). Serum levels of the AHR ligand, indole-3 acetic acid, measured as a predictor of cardiovascular events and mortality in 120 CKD patients with stage 3–5 CKD and stage 5D CKD (Dou et al., 2015). Indole-3 acetic acid (Lin et al., 2019) and another AHR ligand, indoxyl sulfate (Yeh et al., 2016), are additionally associated with cognitive impairments in CKD patients. The gut is the source of indole, which is processed in the liver to indoxyl sulfate, highlighting AHR ligand crosstalk between organs and within the liver (Lowenstein and Nigam, 2021).

The heterogeneity of CKD presentation and progression may be associated with the diversity of AHR ligands that promote and inhibit disease. As shown in Table 1, select AHR compounds that promote kidney injury include non-steroidal anti-inflammatory drugs (NSAIDs), tryptophan metabolites, bacterial pigments, proton pump inhibitors, certain antibiotics, and polycyclic aromatic hydrocarbons (PAHs). Select AHR compounds that inhibit kidney injury include quinoline derivatives, dietary compounds (e.g., resveratrol, indole-3-carbinol), and the tryptophan photo-oxidation product, 6-formylindolo (3,2-b) carbazole (FICZ). The uremic solutes, which include tryptophan metabolites (e.g., indoxyl sulfate, indole-3-acetic acid), contribute to increased vascular permeability, vessel leakage and inflammation (Falconi et al., 2021). The mechanisms may involve indoxyl sulfate-induced endothelial adhesion molecules, which bind to and recruit leukocytes as shown *in vitro* (Ito et al., 2016). In addition, the AHR antagonist, resveratrol, blocked indoxyl sulfate-induced bovine aorta endothelial cell permeability *in vitro* (Assefa et al., 2019), further supporting a function of AHR in CKD pathogenesis.

**TABLE 1:** Function of select AHR ligands in chronic kidney disease.

Molecular class	AHR ligand	Experimental results	Setting	Effect on CKD
Non-steroidal anti-inflammatory drug (NSAID)	**Diclofenac**	Decreased renal perfusion in healthy subjects 1 h after a single oral dose (50 mg) in health subjects	Human	Promotes
	Anti-inflammatory Bass et al. (2009)	Hellms et al. (2019)		
NSAID	**Sulindac**	Promotes chronic decrements in glomerular filtration rate in patients with renal insufficiency Murray et al. (1995)	Human	Promotes
	Anti-inflammatory Ciolino et al. (2006)			
Quinoline-3-carboxamide derivative that is structurally similar to kynurenine	**Laquinimod**	Oral therapy (1–25 mg, x3/week) delays the development of lupus nephritis in a murine model Lourenco et al. (2014)	Mouse	Inhibits
	Anti-inflammatory Blocks S100A9 binding to toll-like receptor (TLR)-4 or receptor for advanced glycation end-products (RAGE) Boros and Vecsei (2020)			
Quinoline-3-carboxamide derivative that is structurally similar to kynurenine	**Paquinimod**	Paquinimod in drinking water inhibits glomeruli complement deposition and hematuria in a murine model (Bengtsson et al., 2012)	Mouse	Inhibits
	Anti-inflammatory Blocks S100A9 binding to TLR4 or RAGE Boros and Vecsei (2020)			
Quinoline derivative that reversibly inhibits ATP binding to vascular endothelial growth factor receptor-2 (VEGR-2)	**Semaxanib**	Intravenous (x2/week, 145 mg/m^2^) administration induced complete resolution of all metastatic tumors in a renal cell carcinoma patient (Jennens et al., 2004)	Human	Inhibits
	Anti-inflammatory VEGFR-2 inhibitor Mezrich et al. (2012)			
4-amino-5-(4-chlorophenyl)-7-(dimethylethyl) pyrazolo [3,4-d]pyrimidine (PP2)]	**PP2**	Intraperitoneal (2 mg/kg) injection improves kidney function and attenuates kidney tubular injury in a murine LPS-induced acute kidney injury model Pak et al. (2020)	Mouse	Inhibits
	Antagonizes proliferation, inflammation, differentiation, adhesion, migration, apoptosis, autophagy and angiogenesis Src family kinase inhibitor Frauenstein et al. (2015)			
Proton pump inhibitor	**Omeprazole**	Promotes dose-dependent cell death in human and murine proximal tubular cell lines and in human primary proximal tubular cell cultures (Fontecha-Barriuso et al., 2020)	Human cell culture	Promotes
	Inhibits parietal cell H+/K + ATP pump Novotna et al. (2014)			
Proton pump inhibitor	**Lansoprazole**	Intraperitoneal (25 mg/kg) injection increases cell death and inflammation in a murine cisplatin-induced acute kidney model Ye et al. (2021)	Mouse	Promotes
	Inhibits parietal cell H+/K + ATP pump Novotna et al. (2014)			
Antibiotic	**Rifampicin**	Acute kidney injury in 25 tuberculosis and leprosy patients in response to rifampicin therapy Muthukumar et al. (2002)	Human	Promotes
	Binds and inhibits bacterial DNA-dependent RNA polymerase Puyskens et al. (2020)			
Tryptophan metabolite	**Kynurenine**	Lower estimated glomerular filtration rate was related to higher plasma kynurenine levels in a meta-analysis Cheng et al. (2020)	Human	Promotes
	Anti-inflammatory Mezrich et al. (2010)			
Tryptophan metabolite	**Indole sulfate**	Induces glomerular lesions in mice, alters podocyte function and increases inflammation Ichii et al. (2014) Levels are associated with increased mortality in hospital-acquired acute kidney injury Wang et al. (2019)	Mouse Human	Promotes
	Pro-inflammatory			
	Uremic toxin Schroeder et al. (2010)			
Tryptophan metabolite	**Indole-3-acetic acid**	Blood levels are increased with chronic kidney disease stage 5D and fell substantially after kidney transplantation Liabeuf et al. (2020)	Human	Promotes
	Pro-inflammatory			
	Pro-thrombotic			
	Uremic toxin Addi et al. (2019)			
Tryptophan metabolite	**Indoxyl glucuronide**	Serum levels are elevated in hemodialysis patients Itoh et al. (2013)	Human	Promotes
	Hypoxic transcription factor antagonist			
	Uremic toxin Asai et al. (2018)			
Tryptophan photo-oxidation product	**6-formylindolo (3,2-b) carbazole (FICZ)**	Intraperitoneally FICZ administered (100 μg/kg/d for 4 days) to mice with rhabdomyolysis and ischemia/reperfusion-induced acute kidney injury attenuated kidney damage Tao et al. (2021)	Mouse	Inhibits
	Promotes IL-22 production			
	Regulates Th17 and T regulatory cell development			
	Concentration-dependent activity Rannug and Rannug (2018)			
Polycyclic aromatic hydrocarbons (PAHs)	**Benzo (a) pyrene (B[a]P)**	Intraperitoneal injection produces oxidative stress, DNA damage and reduced kidney function Deng et al. (2018)	Mouse	Promotes
	Carcinogen Shimizu et al. (2000)			
*Pseudomonas aeruginosa* bacterial pigments	**Phenazines** (1-hydroxyphenazine, phenazine-1-carboxylic acid, phenazine-1-carboxamide, pyocyanin)	*Pseudomonas aeruginosa* urinary tract infections are associated with high mortality in hospitalized patients Lamas Ferreiro et al. (2017)	Human	Promotes
	Pro-inflammatory			
	Cytotoxic Moura-Alves et al. (2014)			
*Mycobacterium tuberculosis* bacterial pigment	**Naphthoquinone phthiocol**	Interstitial nephritis and acute renal failure occur in response to disseminated infection or a localized genitourinary disease Daher Ede et al. (2013)	Human	Promotes
	Pro-inflammatory			
	Cytotoxic Moura-Alves et al. (2014)			
Epstein-Barr virus (EBV) latent protein	**EBV nuclear antigen 3**	EBV genome is present in proximal tubule epithelial cells of patients with chronic interstitial nephritis Becker et al. (1999)	Human	N/A
	Function not significantly explored Kashuba et al. (2006)			
Bioactive compound found in cruciferous vegetables	**Indole-3-carbinol (I3C)**	Oral pre-treatment (20 mg/kg/day) improves cisplatin-induced acute nephrotoxicity indices in rats El-Naga and Mahran (2016)	Rat	Inhibits
	Anti-inflammatory			
	Anti-angiogenic (Popolo et al., 2017)			
Polyphenolic compound present in grapes	**Resveratrol**	Oral administration (5 mg/day/100 g) at the initiation of a rat anti-glomerular basement membrane nephritis model reduces proteinuria, hypoalbuminemia and hyperlipidemia Nihei et al. (2001)	Rat	Inhibits
	Antagonizes AHR transcriptional responses in an estrogen receptor-α-dependent manner			
	Chemoprotective			
	Cardioprotective Perdew et al. (2010)			

Legend. Shown are 12 classes of compounds that bind aryl hydrocarbon receptors (AHR), either activating or suppressing AHR activity. Some compounds affect kidney function. Compounds with negative effects tend to speed chronic kidney disease (CKD) progression, while those with positive effects tend to slow CKD progression in animal models and/or human patients.

Independent of the inciting cause of kidney damage, subsequent progression of CKD is associated with metabolic disturbances, oxidative stress, and inflammation, all of which promote fibrogenesis, irreversible nephron loss, and ultimately reduce the glomerular filtration rate (Ruiz-Ortega et al., 2020). Reduced kidney function leads to the retention of various metabolic products, particularly nitrogenous compounds and often excess fluid, sodium, potassium and phosphate, among many small molecules. These metabolic alterations are characteristic of uremia, and some of these in turn, promote cardiovascular disease (Ghoshal and Freedman, 2019).

## Fibrosis in CKD

Tissue damage from injury or disease induces tissue remodeling and repair. Dysregulation of this process causes an imbalance in extracellular matrix (ECM) homeostasis and the formation of fibrotic tissue. Kidney interstitial ECM consists of diverse molecules, of which the principal families are collagen (types I, II, III, V, VI, VII, and XV), glycoproteins (*e.g.,* fibronectin, laminin), proteoglycans (*e.g.,* biglycan, decorin, versican) and glycosaminoglycans (*e.g.,* chondroitin sulfate, dermatan sulfate, heparin sulfate, hyaluronan). Collectively, these molecules anchor cells within tissue (Bulow and Boor, 2019).

The human genome encodes three isoforms of TGF-β (TGF-β1, TGF-β2, TGF-β3). With regard to extracellular matrix biology; all three isoforms may promote collagen production and tissue fibrosis (Curran and Keely, 2013; Sun T. et al., 2021; Panizo et al., 2021). Activated inflammatory cells secrete inactive TGF-β1 bound non-covalently to a latency associated peptide (LAP), which is disulfide bound to the latent TGF-β binding protein (LTBP). Tissue transglutaminase-2 binds the released complex of LTBP:LAP:TGF-β1 to the ECM by enzymatically cross-linking LTBP to fibronectin and possibly additional ECM proteins associated with elastic fibers (Nunes et al., 1997). Active TGF-β1 is released from the large latent complex by integrin-mediated mechanical deformation of the ECM and/or through degradation of LAP by proteases (*e.g.,* MMPs, thrombospondin-1, and plasmin) (Curran and Keely, 2013).

Intracellular signals downstream from the TGF-β1 receptor stimulate interstitial myofibroblast proliferation and secretion of collagen and additional ECM proteins (Panizo et al., 2021), promote anaerobic metabolism (Zhao et al., 2020), regulate immune cell differentiation (Sanjabi et al., 2017), and induce the expression of integrins (Curran and Keely, 2013) and the nuclear factor-kappa-B (NF-κB) subunit, p65 (Sun et al., 2015). Peroxisome proliferator-activated receptor-gamma (PPAR)-γ ligands (Guo et al., 2004), estrogen (Ito et al., 2010), and AHR ligands (Woeller et al., 2016; Shi et al., 2020) antagonize TGF-β1 cell signaling. The expression of TGF-β1 is induced by hypoxia (Mingyuan et al., 2018), angiotensin II (Kagami et al., 1994), cytokines (interleukin (IL)-4, IL-13), advanced glycation end products (AGEs) (Li et al., 2004), and autocrine cell signals (Bhogal et al., 2005). These factors are therefore likely to contribute to fibrogenesis in CKD.

With respect to AHR, the quinoline-3-carboxamide derivative and AHR ligand, paquinimod, inhibits fibrosis in murine models of experimental systemic sclerosis (Stenstrom et al., 2016) and liver fibrosis (Fransen Pettersson et al., 2018). The anti-fibrotic functions of paquinimod in CKD have not been significantly explored. Mice fed a diet supplemented with 0.25% adenine generate increased levels of the AHR ligand, indoxyl sulfate, comparable to human CKD patients, leading to periglomerular fibrosis (Walker et al., 2020). These data are supported in rats administered 200 mg/kg indoxyl sulfate drinking water, resulting in the elevated expression of the mesenchymal marker, α-smooth muscle actin, and increased Masson’s trichrome-positive fibrosis in the kidney (Bolati et al., 2011). Indoxyl sulfate induces TGF-β1 expression and production in human proximal tubular cells (HK-2 cells) and the antioxidant, indole-3-propionic acid, suppresses this response (Yisireyili et al., 2017). The indole acetic acid derivative, mitochonic acid 5 (MA-5), inhibits mitochondrial reactive oxygen species and improves renal function in an ischemia-reperfusion injury model and a cisplatin–induced nephropathy model (Suzuki et al., 2016). Moreover, mice administered MA-5 through osmotic pump in a model of unilateral ureteral obstruction demonstrated reduced expression of TGF-β1, decreased collagen I staining, and reduced renal fibrosis (Shima et al., 2017). Because dioxin-induced AHR promotes mitochondrial reactive oxygen species production and in AHR deficient mice, cellular mitochondrial reactive oxygen species are lower compared to controls (Brinkmann et al., 2019), the type of ligand and presence of AHR are likely important to oxidative stress in CKD.

Oxidative stress is associated with the activity of lysyl oxidases (LOX) (Martinez-Revelles et al., 2017) and transglutaminases (Basso et al., 2012), which promote vascular stiffness and neuronal death in murine models, respectively. These pleotropic proteins are involved in cell signaling and post-translational modifications, including the cross-linking of collagen. Calcium-dependent transglutaminases catalyze protein cross-links by introducing glutamyl-lysyl isopeptide bonds between target proteins. LOX catalyzes the formation of aldehydes from lysine residues in collagen and elastin, and this promotes cross-linking of these molecules in tissue (Heck et al., 2013). Transglutaminase-2 and LOX expression are induced by hypoxia and TGF-β (Curran and Keely, 2013), indicating that these molecules facilitate increased cross-linking and fibrosis in tissues with a compromised blood supply.

Excessive ECM accumulation is prevented by the activity of matrix metalloproteinases (MMPs), which cleave fibrillar collagens (Zhao et al., 2020). The AHR ligands FICZ (Shi et al., 2020), kynurenine (Li et al., 2014) and dioxin (Tsai et al., 2014) promote MMP-1 *in vitro.* The AHR ligand indole-3-carbinol inhibits MMP-2 and MMP-9 *in vitro* (Wu et al., 2005) and in a murine model of liver fibrosis, the AHR ligand paquinimod reduced the expression of MMP-2 (Fransen Pettersson et al., 2018), highlighting the diversity of the AHR response in ECM maintenance. Increased production and deposition of type I collagen, primarily by fibroblasts, is associated with increased transglutaminase-2 activity, promoting the formation of a stiff matrix; this can progress to overt kidney fibrosis (Bulow and Boor, 2019; Zhao et al., 2020).

In a study of 202 kidney disease cases of different etiologies, the levels of serum LOX and tissue LOX in renal biopsies were associated with the presence and degree of kidney fibrosis across diseases (Zhang XQ. et al., 2020). In cyclosporin A-induced nephropathy in mice, treatment with LOX inhibitors attenuated inflammation, fibrosis and uremia (Nguyen et al., 2021). Additionally, rats undergoing 5/6-nephrectomy and treated with transglutaminase inhibitors prevented a decline in kidney function and interstitial fibrosis (Johnson et al., 2007), highlighting these cross-linkers as targets in CKD. A role for AHR in regulating these cross-linkers through MMP production or oxidative responses has not been significantly explored.

## CKD Therapies

Treatment of CKD involves several approaches. Treating the underlying disease, including systemic diseases (diabetes mellitus, systemic lupus, systemic vasculitis, and others), may slow or halt progression (Moiseev et al., 2020; Navaneethan et al., 2021). For example, the sodium-glucose transport protein 2 (SGLT2) inhibitor dapgliflozin was recently approved for progressive non-diabetic kidney disease of many etiologies as a result of studies demonstrating efficacy in reducing the risk for a combined endpoint of kidney function decline, kidney failure, cardiovascular death and hospitalization for heart failure (Wheeler et al., 2021). Pathway-targeted treatments are increasingly available or in development for primary renal diseases or syndromes (*e.g.,* focal segmental glomerulosclerosis, membranous nephropathy, and polycystic kidney disease) (Curran and Kopp, 2021; Finnigan and Leslie, 2021). At the other end of the frequency scale, there are an estimated 10,000 rare diseases (Haendel et al., 2020) and an unknown fraction of these have renal manifestations; many of these lack targeted therapies.

Non-specific therapies for CKD can slow and even halt progressive loss of kidney function. These include diet and medications for blood pressure control (target <130/80) (Faqah and Jafar, 2011), antifibrotic therapies (inhibitors of renin, angiotensin II, and aldosterone, the latter being a potent profibrotic molecule) (Zhang et al., 2019), and dietary sodium restriction and thiazide diuretics (the latter potentiates the antiproteinuric effects of the renin-angiotensin-aldosterone system antagonists) (Park et al., 2014). SGLT2 inhibitors reduce progression of kidney damage in diabetes (Tuttle et al., 2021) and recently this effect has been shown in non-diabetic kidney disease as well, possibly by reducing proximal tubule stress (Almaimani et al., 2021).

There continues to be considerable interest in developing novel anti-fibrotic therapies for kidney disease, although progress has been slow. Pirfenidone is an anti-fibrotic agent (2014 U.S. FDA approval reference ID: 3642437) that showed efficacy in phase two trials for idiopathic pulmonary fibrosis (Raghu et al., 1999; Maher et al., 2020). Preliminary studies of pirfenidone in studies of focal segmental glomerulosclerosis (Cho et al., 2007) and in diabetic nephropathy (Sharma et al., 2011) were encouraging but further development appears to have stalled. The exact mechanisms of action of pirfenidone are not fully characterized. Recent studies suggest the molecule may be a ligand for peroxisome proliferator-activated receptors (e.g., PPAR-α, PPAR-γ) (Hamidi et al., 2021), which appears to inhibit transforming growth factor (TGF)-β1-induced collagen I production (Cui et al., 2020). Pirfenidone may also suppress other profibrotic and pro-inflammatory mediators, including fibroblast growth factor (FGF), platelet-derived growth factor (PDGF), vascular endothelial growth factor (VEGF) (Chaudhary et al., 2007), interleukin-1β (IL-1β) and tumor necrosis factor (TNF) (Evani et al., 2020).

Similarly, ligands of the aryl hydrocarbon receptor (AHR), specifically tryptophan metabolites [*e.g.,* 2-(10 H-indole30-carbonyl)-thiazole-4-carboxylic acid methyl ester (ITE) and FICZ], are capable of inhibiting TGF-β1-induced collagen I production in models of systemic sclerosis and thyroid eye disease (Woeller et al., 2016; Shi et al., 2020). An analog (SZR72) of kynurenic acid, a tryptophan metabolite and AHR ligand, inhibits the production of TNF in human blood cultures (Balog et al., 2021). Also, the plant-derived AHR ligand, indole-3-carbinol (I3C), antagonizes IL-1β production in cell lines (Jiang et al., 2013). AHR expression is induced by growth factors (PDGF, FGF) (Vaziri et al., 1996) and in a murine model of cardiac hypertrophy, AHR antagonizes hypoxia-induced VEGF production and the development of fibrosis (Ichihara et al., 2019). These functions of AHR suggest that AHR may be a therapeutic target in CKD. However, the AHR response is defined by a diverse array of toxins, endogenous molecules, drugs, dietary components, and pathogens that may promote or inhibit CKD (Table 1). AHR may additionally exhibit crosstalk with estrogen receptors, PPAR-γ, NF-kB, and cell signals in hypoxia and TGF-β1 pathways. In this regard, AHR has shown considerable, and puzzling, diversity of function in the kidney and manifestations associated with CKD. The effects of AHR in cell signaling pathways that influence fibrosis, the renin angiotensin aldosterone system (RAAS), and metabolism are therefore herein described.

## AHR Stability, Signaling, and E3 Ubiquitin Ligase Activity

AHR is a ligand-activated transcription factor and E3 ubiquitin ligase with pleotropic functions in mammalian biology. Sequence homology between circadian [period (*PER*)] and neurodevelopment (single-minded (*SIM*) genes in *Drosophila melanogaster* and a gene in the human dioxin signaling pathway (AHR nuclear translocator (*ARNT*)) established the PER-ARNT-SIM (PAS) domain protein superfamily, of which AHR is a member. PAS domains mediate protein-protein and small-molecule-protein interactions. These include AHR dimerization with ARNT and AHR binding interactions with chaperone proteins and ligands (McIntosh et al., 2010).

Inactive AHR exists in the cytosol in a multi-protein complex, which includes a heat shock protein-90 (Hsp90) dimer, a co-chaperone (p23), an AHR interacting protein (AIP/XAP2/ARA9), and various co-activators, including members of Src family of non-receptor tyrosine kinases (Avilla et al., 2020). AIP (Lees et al., 2003) and p23 (Pappas et al., 2018) block ubiquitination of AHR, possibly by inhibiting AHR binding interactions with the E3 ubiquitin ligase C-terminal hsp70-interacting protein (STUB1/CHIP) (Morales and Perdew, 2007). E3 ubiquitin ligases provide a platform for ubiquitin enzymes to transfer ubiquitin to proteins. Ubiquitin binding can either alter protein signaling or target the protein to the proteasome for degradation (Buetow and Huang, 2016). Both ligand-bound and unliganded AHR can be targeted to the proteasome for degradation (Ma and Baldwin, 2000; Morales and Perdew, 2007).

**FIGURE 1:**
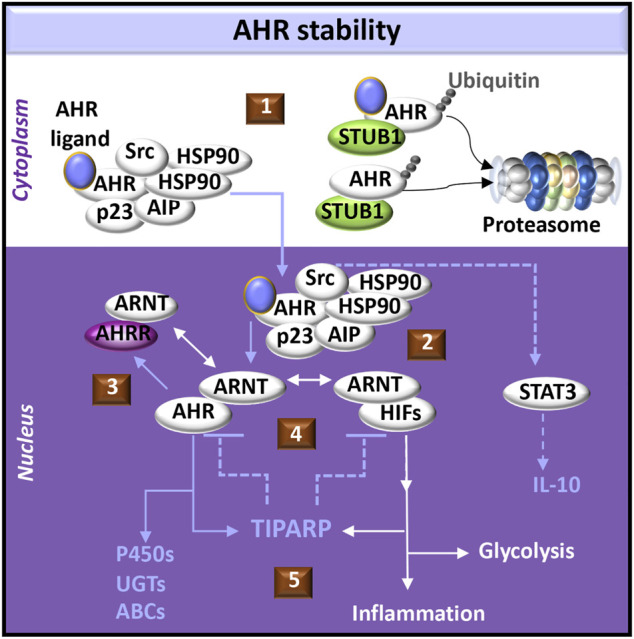
AHR-initiated cell signaling pathways. (1) The aryl hydrocarbon receptor (AHR) forms a complex with chaperone molecules in the cytoplasm. Ligand binding may promote transport of the complex into the nucleus. Alternatively, release of AHR from the complex may promote interactions with an E3 ligase (*e.g.,* STUB1) which acts as a platform for AHR ubiquitination and targeted degradation *via* the proteasome. Both ligand-bound and unliganded AHR can be targeted to the proteasome. (2) AHR dissociates from its cytoplasmic complex to bind the AHR nuclear translocator (ARNT), which alternatively may dimerize with hypoxia inducible transcription factors (HIFs). AHR induces activation of signal-transducer and activator of transcription (STAT3) through Src signaling, acting as a tyrosine protein kinase involved in the production of IL-10. (3) AHR promotes the expression of the AHR repressor (AHRR), which also dimerizes with ARNT and negatively regulates AHR functions by competing with AHR binding sites in DNA regulatory sequences. (4) Activation of AHR or HIFs induces transcription and translation of PARP7 (poly ADP-ribosyl transferase 7), also known as TIPARP (TCDD-inducible poly-ADP-ribose polymerase). TIPARP further promotes polyADP-ribosylation and subsequent degradation of AHR and HIFs (5) Downstream responses of AHR can include activation of the biotransformation enzymes, including cytochrome P450 enzymes (P450s), UDP-gluconosyltransferases (UGTs), and ATP-binding cassette transporters (ABCs). Downstream responses of HIFs can include increased glycolysis and the expression of immunomodulatory genes that provoke inflammation.

Ligand binding to AHR in the multi-protein complex induces conformational changes in AHR and reorganization of the chaperones to facilitate nuclear localization of the complex (Avilla et al., 2020). AHR activation induces Src kinase phosphorylation (Xie et al., 2012) and cell signals that promote the production of IL-10 (Zhu et al., 2018). Release of active AHR from the complex allows AHR to partner with ARNT, which can also either dimerize with and activate hypoxia inducible transcription factors (HIFs) or the AHR repressor (AHRR). HIFs compete with AHR for ARNT dimerization and promote transcription of genes expressing enzymes in the glycolytic pathway (Goda and Kanai, 2012). AHR-induced AHRR not only competes with AHR for ARNT but also suppresses AHR function as a transcription factor by binding AHR responsive elements (AHREs) (Avilla et al., 2020) (Figure 1).

## AHR in Drug Metabolism

**FIGURE 2:**
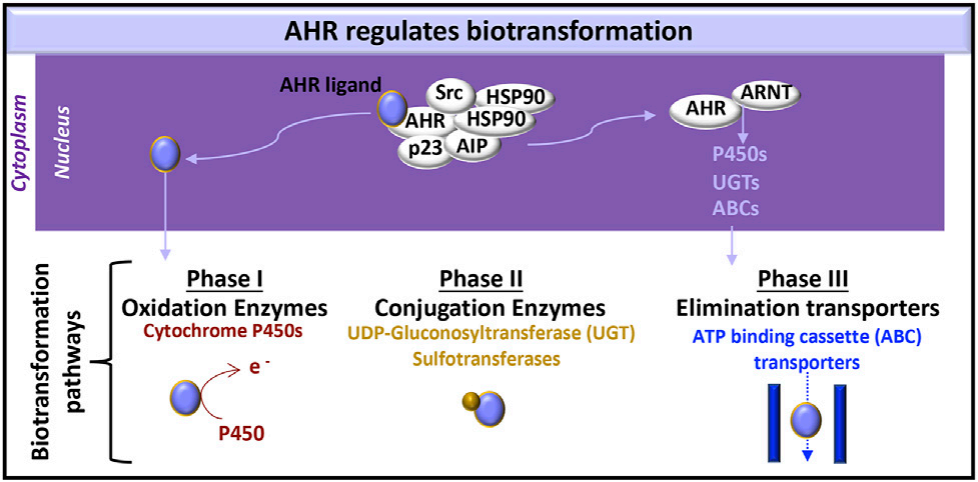
AHR-initiated biotransformation. AHR induces the transcription of certain genes whose products are involved in each of the three phases of drug metabolism.

AHR has a pivotal role in regulating the clearance of xenobiotics and particular endogenous compounds (*e.g.,* metabolites of tryptophan, arachidonic acid and hemoglobin), which also bind and activate AHR during various processes, including development (Avilla et al., 2020), hematopoiesis (Angelos and Kaufman, 2018) and disease pathogenesis (Curran et al., 2018; Curran et al., 2019). In the clearance of these molecules, the AHR:ARNT heterodimer induces the expression of proteins involved each of the three metabolic biotransformation pathways. These proteins neutralize the activity of endogenous and xenobiotic molecules and promote the efflux of these molecules from the cell (Figure 2).

Phase I metabolism includes oxidation, reduction and hydrolysis of substrates, to generate more water-soluble, but generally still active, xenobiotic molecules (Phang-Lyn and Llerena, 2021). AHR-induced cytochrome P450 enzymes are a common measure of AHR activation in the liver. Their relevance in the kidney (Knights et al., 2013) and the immune system (Effner et al., 2017) are subjects of on-going study. Cytochrome P450 enzymes oxidize substrates and therefore contribute to phase I metabolism (Zanger and Schwab, 2013).

Phase II metabolism is carried out by conjugating enzymes, which add a hydrophilic group to targeted molecules; these protein modifications include glucuronidation, sulfation, acetylation, and methylation (Phang-Lyn and Llerena, 2021). UDP-gluconosyltransferase (UGT)-1A5, UTG1A6, UGT1A7, UGT1A9, UGT2B4, UGT2B4, and UGT2B17 are expressed in the kidney (Knights et al., 2013). AHR can induce UGT1A1 (Yueh et al., 2005) and UGT1A6 (Bock and Bock-Hennig, 2010) in human cell lines.

Phase III metabolism includes ATP-binding cassette (ABC) and solute carrier (SLC) transporters that facilitate xenobiotic efflux (Phang-Lyn and Llerena, 2021). Exposing rat brain capillaries to the AHR ligand, *2,3,7,8-tetrachlorodibenzo-p-dioxin* (TCDD, dioxin), induced production of the ABC transporter, P-glycoprotein, also known as multidrug resistance protein (MRP)-1 (Wang et al., 2011). P-glycoprotein and additional ABC transporters [*e.g.,* MRP2, MRP4, breast cancer resistance protein (BCRP)] are also induced in dioxin exposed killifish renal proximal tubules (Mahringer et al., 2019). Further testing of these responses in human kidney cells would enhance our knowledge of AHR kidney function.

## AHR Function in ADP-Ribosylation

Both activated AHR (MacPherson et al., 2013) and HIF-1α (Zhang L. et al., 2020) promote transcription of the *TCDD-inducible poly(ADP-ribose) polymerase (TIPARP/PARP7/ARTD14)* gene. Poly-ADP-ribose polymerases (PARPs) post-translationally add a single ADP-ribosyl group (mono-ADP-ribosylation/MARylation) or multiple groups (poly-ADP-ribosylation/PARylation) to substrates (*e.g*., protein, DNA, and RNA) in regulating DNA repair, transcription, and cell signaling (Sanderson and Cohen, 2020). While the functional attributes of PARPs continue to be characterized, TIPARP uniquely is capable of negatively regulating its transcriptional activators (AHR, HIF-1α) (Figure 1). The mechanisms appear to involve TIPARP co-localization with the particular transcription factor and the recruitment of an E3 ligase for ubiquitin-mediated proteasome degradation of the transcription factor (MacPherson et al., 2013; Zhang L. et al., 2020). These integrated networks involving AHR and HIFs implicate AHR in basic metabolic processes and hypoxic responses during the development and progression of diseases such as CKD (Fu et al., 2016).

## AHR Regulatory Mechanisms in CKD Signaling Networks

**FIGURE 3:**
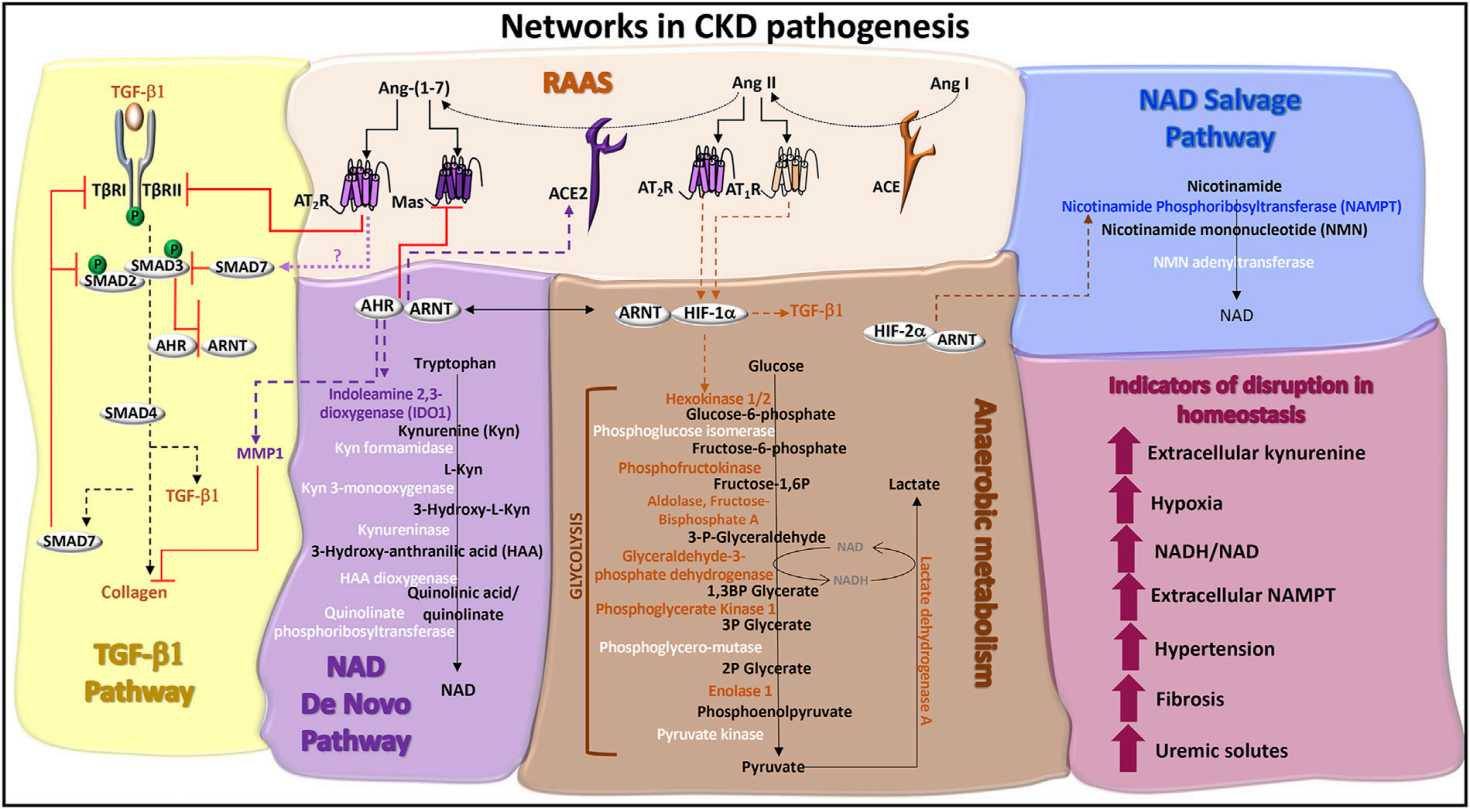
Networks in CKD pathogenesis. **TGF-β1 pathway:** TGF-β1 signaling is initiated through serine/threonine kinase receptors, TGF-β1 receptor (TβR)-I and TβRII. TGF-β binding to TβRII recruits TβRI to form a receptor heterodimer, which is phosphorylated. SMAD2 and SMAD3 are recruited to the receptor heterodimer and are also phosphorylated. SMAD2 and SMAD3 co-localize with SMAD4 and translocate to the nucleus to activate genes, such as collagen and TGF-β1. ACE2 activity promotes the production of SMAD7, potentially *via* Ang-(1–7)-induced Mas or AT_2_R receptor signals. SMAD7 is a negative regulator of TGF-β1 by recruiting E3 ligases to TβRI and blocking TβRI-induced SMAD2/3 phosphorylation. Ligand activated AHR antagonizes TGF-β1 and collagen gene expression and protein production, which are associated with fibrosis. Ligand activated AHR also induces the degradation of collagen through the production of matrix metalloproteinase-1 (MMP1). **Renin angiotensin aldosterone system (RAAS):** Angiotensin I (Ang I) is cleaved by angiotensin converting enzyme (ACE) into Ang II. The binding of Ang II to AT_1_R or AT_2_R promotes the stable expression of HIF-1α and hypoxic responses. The ACE homolog, ACE2, inactivates Ang II by cleaving and processing Ang I and Ang II into Ang-(1–7), which is a ligand for the Mas receptor and AT_2_R. AHR regulates the expression of ACE2 and Mas. AT_2_R activation promotes TβRII degradation, inhibiting TGF-β1 signals. **NAD de novo biosynthesis pathway (also known as the kynurenine pathway):** Tryptophan catabolism is the defining feature of this pathway. The rate limiting enzymes are indoleamine dioxygenase (IDO) and tryptophan dioxygenase (TDO). IDO1 is induced by AHR:ARNT transcriptional activation of the IDO promoter and promotes the production of kynurenine, which can be released as a cytokine. A series of additional enzymes (highlighted in white) catalyze the production of immunomodulatory and neuroregulatory molecules that are further processed into NAD. **Anaerobic metabolism:** The production of adenosine triphosphate (ATP) in the absence of oxygen occurs through enzymatic reactions in glycolysis and results in the production of lactate and the increased formation of NADH relative to NAD. Enzymes in this pathway are regulated by HIF-1α activated genes (highlighted in orange), which can be stabilized by the RAAS. **NAD salvage pathway:** The primary source of mammalian NAD is from the recycling nicotinamide, which is the amide version of vitamin B3 and a by-product from the enzymatic activity of Poly-ADP-ribose polymerases (PARPs) and sirtuins. The rate limiting enzyme is nicotinamide phosphoribosyltransferase (NAMPT), which is transcriptionally activated by HIF-2α and functions as an extracellular cytokine. **Indicators of disruption in homeostasis:** Factors and conditions along these pathways are induced during CKD pathogenesis.

CKD manifests hypertension (Panizo et al., 2021), progressive kidney fibrosis (Bulow and Boor, 2019), and tissue hypoxia (Fu et al., 2016). Biochemical features include increased circulating plasma levels of extracellular nicotinamide phosphoribosyltransferase (eNAMPT/visfatin) (Hsu et al., 2016) and elevated blood levels of kynurenine (Cheng et al., 2020) (Figure 3). These pathologic processes and biomarkers are regulated by the RAAS, TGF-β1 cell signals, and metabolism. The roles of AHR in each of these systems is yet to be fully characterized.

## RAAS and AHR

The RAAS pathway involves a series of enzymatic reactions that contribute to the homeostatic control of extracellular fluid volume, arterial pressure, tissue perfusion, electrolyte balance, and wound healing. Renin, released from glomerular juxtaglomerular cells, processes liver-produced angiotensinogen into angiotensin I (Ang I), which is further cleaved by a soluble ectoprotein, angiotensin converting enzyme (ACE), into Ang II. The binding of Ang II to the Ang II type 1 receptor (AT1R) promotes vasoconstriction and also induces the production of aldosterone, which promotes renal tubular sodium reabsorption and promotes fibrogenesis. The ACE homolog, ACE2, inactivates Ang II by cleaving and processing Ang I and Ang II into Ang (1–7), which binds the Mas receptor and the AT_2_R. By these activities, ACE2 antagonizes the vasoconstrictive, inflammatory, prothrombotic, and fibrotic effects associated with ACE/Ang II/AT_1_R activity (Curran et al., 2020).

The functions of AHR in the RAAS signaling pathways appear to depend upon the level of expression of AHR and the AHR ligand. AHR-deficient mice develop cardiac hypertrophy through mechanisms involving HIF-1α cell signals (Thackaberry et al., 2002), Ang II-induced fibrosis (Ichihara et al., 2019), increased plasma levels of endothelin-1, and elevated mean arterial pressures (Lund et al., 2003). In heterozygous AHR (+/−) mice, blood pressure remains normal, and AHR (+/−) mice are more responsive to ACE inhibition and an endothelin-1 receptor antagonist compared to AHR (−/−) null mice (Zhang et al., 2010).

**FIGURE 4:**
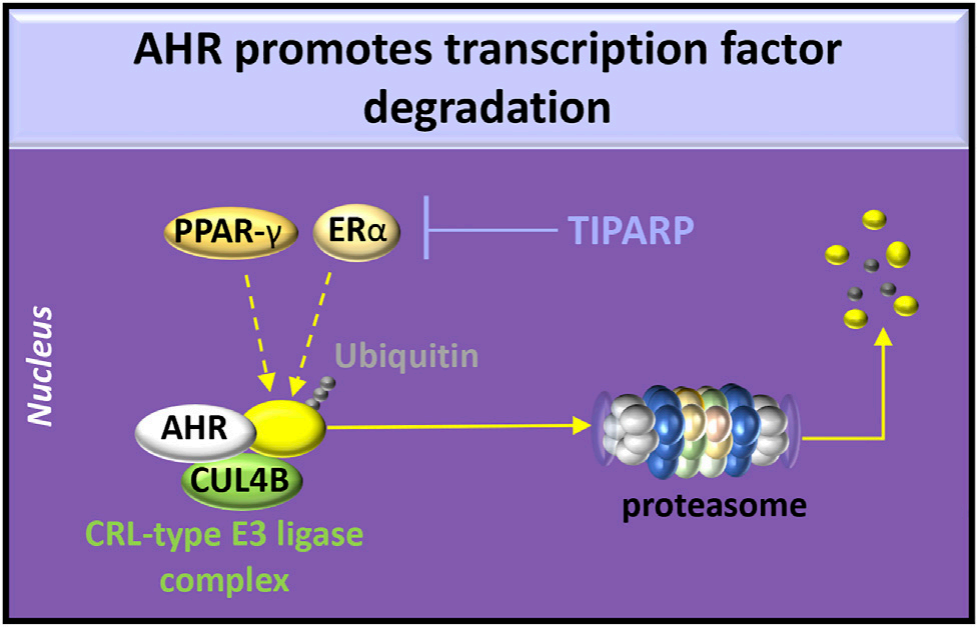
AHR degradative functions. AHR participates in the cullin/RING ubiquitin ligase (CRL-type E3 ligase) complex involving chaperones [*e.g.*, cullin 4b (CUL4B)] to promote ubiquitination of estrogen receptor (ER)-α or peroxisome proliferator-activated receptor (PPAR)-γ. Activation of AHR or HIFs induces transcription and translation of PARP7 (poly ADP-ribosyl transferase 7), also known as TIPARP (TCDD-inducible poly-ADP-ribose polymerase). TIPARP promotes polyADP-ribosylation and subsequent degradation of ER-α.

The effects of overexpression of AHR on RAAS activity have not been extensively examined. AHR overexpression in adipocytes shortens the half-life of PPAR-γ by recruiting PPAR-γ to the cullin 4b (CUL4B)-RING E3 ubiquitin ligase complex (Dou et al., 2019), leading to PPAR-γ degradation in the proteasome. Because reduced PPAR-γ activity induces AT_1_ expression and signaling in human fibroblasts (Auclair et al., 2013), overexpression of AHR may also affect the ACE/Ang II/AT_1_R pathway. Estrogen receptor (ER)-α can also be recruited by AHR to the CUL4B-RING E3 ubiquitin ligase complex resulting in ubiquitination (Luecke-Johansson et al., 2017) or targeted by TIPARP ADP-ribosylation (Zhang L. et al., 2020). These post-translational modifications that promote ER-α proteasome degradation indicate a potential role of AHR in sex-related differences in RAAS activity (Sabbatini and Kararigas, 2020) (Figure 4).

Various AHR ligands increase in abundance during CKD pathogenesis and may affect RAAS activity. Reduced kidney function causes blood retention of a heterogeneous mix of metabolites (uremic solutes), which may be more effectively removed by continuous ambulatory peritoneal dialysis compared to low-flux hemodialysis (Xie et al., 2019). Tryptophan metabolites are uremic solutes and are AHR ligands, which promote CKD progression (Table 1). In a small study of CKD patients, an increased ratio of the tryptophan metabolite, kynurenine, to tryptophan was associated with macroalbuminuria and responsiveness to AT_1_R blockers (ARBs) (Wu et al., 2020). This study suggests upregulation of the ACE/Ang II/AT_1_R pathway in these individuals. However, *in vitro* examination of kynurenine-exposed BEAS-2B lung epithelial cells revealed AHR-induced production of ACE2 (Lv et al., 2021).

Because ACE2 activity antagonizes ACE/Ang II/AT_1_R cell signaling (Curran et al., 2020), additional factors in the RAAS pathway may be affected by AHR. One such factor is the Mas receptor. In normotensive and hypertensive rats, oral administration of the uremic solute, indoxyl sulfate, reduced kidney Mas receptor expression. This study further demonstrated that indoxyl sulfate-exposed human kidney-2 (HK-2) proximal tubular cells reduced Mas receptor expression and this response was antagonized by Ang-(1–7) pre-treatment or by the absence of AHR (Ng et al., 2014). Thus, AHR expression and activation are integral to the RAAS homeostatic function.

## Metabolism and AHR

Ischemia and oxidative stress induce metabolic stressors (*e.g.,* hypoxia) and the production of pro-inflammatory mediators (e.g., IL-1β, TNF, IL-6) during the progression of CKD. In a comprehensive pathway map analysis of gene sets from 157 European patients with nine different types of CKD, metabolism and inflammation were identified as the two main pathways in the pathology leading to CKD progression (Stenvinkel et al., 2021). Transcription factors, such as NF-κB and HIFs, regulate these responses and exhibit crosstalk with AHR.

There are three isoforms of HIF-α: HIF-1α, HIF-2α, and HIF-3α, encoded by distinct genes: *HIF1A*, *EPAS1*, *HIF3A*. HIF-1α and HIF-2α are expressed in the kidney, compete with AHR for the dimer partner ARNT, and can be degraded in either the proteasome or the lysosome (Hubbi et al., 2013; Jochmanova et al., 2013). Oxidative stress, hypoxia, Ang II, and certain peptides (*e.g.*, human epidermal growth factor receptor-2 (HER-2), IL-1β, insulin) (Wolf, 2005; Curran and Keely, 2013) reduce HIF post-translational modifications that target the protein for degradation and thereby stabilize HIF expression.

The most well-described HIF-1α transcriptional responses involve the expression of genes whose products are involved in glycolysis and the production of lactate (Figure 3). Specifically, the HIF-1α: ARNT heterodimer promotes transcription of *SLC2A1* (Solute Carrier Family two Member 1, GLUT1), *HK1* (hexokinase 1), *HK2* (hexokinase 2), *PFK* (phosphofructokinase), *ALDOA* (aldolase, fructose-bisphosphate A), *GAPDH* (glyceraldehyde-3-phosphate dehydrogenase), *PGK1* (phosphoglycerate kinase 1), and *LDHA* (lactate dehydrogenase A) (Semenza et al., 1996; Jochmanova et al., 2013; Del Rey et al., 2017). Anaerobic metabolism decreases the levels of nicotinamide adenine dinucleotide (NAD) and increases the formation of the reduced form, NADH (Figure 3). Despite their differential functions in the RAAS, AT_1_R and AT_2_R can both induce HIF-1α stabilization in response to Ang II (Wolf, 2005; Liu et al., 2014). Understanding the roles of AT_1_R and AT_2_R HIF-1α cell signals in CKD requires further study.

The von Hippel-Lindau tumor suppressor (VHL) is an E3 Ubiquitin Ligase Which Ubiquitylates and targets the α-subunit of HIFs for oxygen-dependent proteolysis (Haase, 2009). Deletion of the *VHL* gene or increased production of HIF-2α in mouse podocytes leads to rapidly progressive glomerulonephritis that can be prevented by targeted deletion of the *ARNT* gene (Ding et al., 2013). Understanding whether AHR functions in the absence of ARNT in these cells may not only offer insight to the functions of podocytes, but also mechanisms in NAD metabolism, particularly since ARNT is a factor in both NAD salvage and *de novo* biosynthesis pathways.

The HIF-2α: ARNT heterodimer activates the transcription of nicotinamide phosphoribosyltransferase (NAMPT/visfatin) (Sun et al., 2020), the rate limiting enzyme in the NAD salvage pathway (Garten et al., 2015). Plasma levels of NAMPT are negatively correlated with glomerular filtration rate in nondiabetic hypertensive patients (Hsu et al., 2016), chronic glomerulonephritis patients, and diabetic nephropathy patients (Axelsson et al., 2007). As a cytokine, NAMPT promotes the production of inflammatory mediators, upregulates the expression of adhesion receptors and induces endothelial dysfunction (Romacho et al., 2013). Inside the cell, NAMPT catalyzes the production of nicotinamide mononucleotide (NMN) (Garten et al., 2015) (Figure 3). In HK-2 cells exposed to 1 mM hydrogen peroxide plus 1% oxygen, NMN increased cell viability and reduced collagen IV protein production. Similar results were obtained in a murine ischemia-reperfusion injury model, manifesting reduced tubular DNA damage, cellular injury, and fibrosis in response to intraperitoneal NMN therapy (Jia et al., 2021). These data suggest that neutralizing extracellular NAMPT or activating intracellular NAMPT production of NMN may be therapeutic strategies in treating CKD.

The AHR: ARNT heterodimer promotes the transcription of indoleamine 2,3- dioxygenase (IDO1) (Bessede et al., 2014), the rate limiting enzyme in the NAD *de novo* biosynthesis pathway. This pathway catabolizes tryptophan into NAD through a series of enzymatic reactions, which are also part of the kynurenine pathway (Ralto et al., 2020) (Figure 3). In a small study of CKD patients, plasma IDO1 activity level and its downstream metabolites (kynurenine, kynurenic-acid, quinolinic-acid) correlated with disease severity (Schefold et al., 2009). This finding concurs with conclusions from a subsequent study correlating serum IDO1 and kynurenine levels with disease severity (Bao et al., 2013). A meta-analysis indicates that the lower estimated glomerular filtration rate in these patients is associated with higher blood metabolite levels of tryptophan metabolites, including kynurenine, C-glycosyltryptophan (glycosylated amino acid), 3-indoxyl sulfate, and indole-3-lactate (Cheng et al., 2020). Each of these studies demonstrate an increase in the production of AHR ligands and a decrease in the production of NAD from the *de novo* biosynthesis pathway.

Quinolinate phosphoribosyltransferase (QPRT) is the final enzyme in the *de novo* biosynthesis pathway (Figure 3). In QPRT^+/−^mice, lower levels of NAD in the kidney and higher urinary levels of quinolinate are identified. In response to renal ischemia-reperfusion injury in wild-type mice, kidney QRPT expression levels are reduced and urinary quinolinate levels are elevated. These models were supplemented with intraperitoneal injections of 400 mg/kg nicotinamide, which ameliorated kidney function (Poyan Mehr et al., 2018), presumably through the production of NAD in the salvage pathway (Figure 3). In a small study of patients with COVID-19-related acute kidney injury, 1 g oral nicotinamide/day over 7 days, reduced mortality and renal replacement therapy, highlighting a potential therapeutic function in promoting NAD via the salvage pathway (Raines et al., 2021). Because a primary AHR-induced gene and regulator, TIPARP, also requires NAD for full activation (Zhang L. et al., 2020), further exploration of TIPARP in CKD progression may provide insight into the therapeutic functions of intracellular NAD.

## The TGF-β1 Pathway and AHR

AHR is an integral regulator of extracellular matrix assembly and remodeling. AHR competition with HIF-1α for ARNT may be a mechanism which antagonizes three pro-fibrotic processes: hypoxia induction of TGF-β1, leading to suppressed collagen expression (Mingyuan et al., 2018); hypoxia-induced collagen prolyl hydroxylases (P4HA1 and P4HA2) required for collagen maturation and deposition (Gilkes et al., 2013); and hypoxia-induced enzymes (*e.g.*, LOX, transglutaminases, lysyl hydroxylases) involved in collagen cross-linking (Curran and Keely, 2013; Gilkes et al., 2013). The mechanisms by which ligand activated AHR inhibits alpha-smooth muscle actin and collagen I expression and promotes MMP-1 expression are not fully known but may involve AHR crosstalk with various signaling pathways (Poormasjedi-Meibod et al., 2016; Shi et al., 2020).

Two key signaling pathways in renal fibrosis include the TGF-β1 pathway and the NF-κB pathway, which can also exhibit crosstalk between each other and with AHR. TGF-β1, β2 and β3 initiate cell signals through activation of TGF-β receptor (TβR)-I and TβRII heterodimers. TβRII phosphorylates TβRI, which recruits and phosphorylates signaling transducer molecules. These receptor-regulated SMADs (*e.g.,* SMAD2, SMAD3) co-localize with the common partner SMAD (SMAD4) prior to localizing to the nucleus and binding to target genes (Chen et al., 2018). SMAD3 also strongly interacts with both AHR and ARNT and promotes dissociation between AHR and ARNT, inhibiting AHR signals (Nakano et al., 2020).

SMAD7 is an inhibitor SMAD that is activated by TGF-β signaling, providing negative-feedback. SMAD7 is recruited to TβRI and prevents receptor-regulated SMAD docking and phosphorylation. SMAD7 also recruits E3 ubiquitin ligases to degrade TβRI, SMAD2 and SMAD3, creating a regulatory feedback loop in TGF-β signaling (Chen et al., 2018).

The RAAS pathway is integrated with the TGF-β cell signal pathway. In an animal model involving Ang II-induced renal fibrosis, Smad7 knockout mice exhibit elevated TGF-β/Smad3 signaling, more severe renal injury and increased progressive fibrosis compared to the wild-type (Liu et al., 2013). However, in Smad3 knockout mice, Ang-II-induced renal fibrosis and NF-κB-driven renal inflammation was significantly lower compared to the wild-type (Liu et al., 2012b), highlighting functions of Ang II in promoting TGF-β cell signals.

Moreover, in mice with unilateral ureteral obstruction nephropathy, deletion of ACE2 results in a fourfold increase in the ratio of intrarenal Ang II/Ang 1-7, which was associated with increased tubulointerstitial fibrosis and inflammation (Liu et al., 2012a). Subsequent mechanistic studies in Ace2 knockout mice subjected to chronic subcutaneous angiotensin II infusion revealed an increase in Smad-specific E3 ubiquitin protein ligase 2 (SMURF2), a decrease in renal SMAD7, and increased TGF-β and NF-κB (Liu et al., 2017).

Because ACE2 is not known to directly transduce signals, a product of ACE2 activity, Ang (1–7), may function in the regulation of TGF-β cell signaling. Ang (1–7) binds the AT_2_R and the Mas receptor (Curran et al., 2020). The AT_2_R agonist, CGP42112A, promotes AT_2_R co-localization with TβRII for subsequent TβRII degradation (Guo et al., 2016). Possibly, Ang (1–7) activation of AT_2_R also induces the production of SMAD7. Because AHR induces ACE2 production (Lv et al., 2021) AHR may play a role in each of these responses (Figure 3).

## The NF-KB Pathway and AHR

**FIGURE 5:**
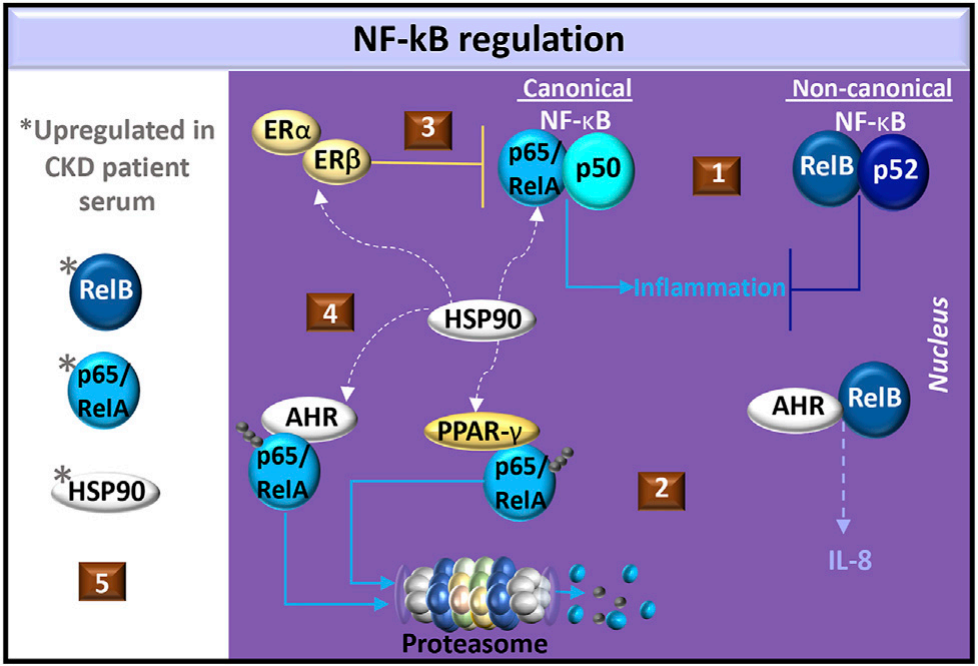
Regulation of NF-κB in CKD. (1) Common NF-κB dimer pairs include p65/RelA and p50 in the canonical pathway and RelB and p52 in the non-canonical pathway. Non-canonical cell signals may antagonize canonical cell signals in fibroblasts. (2) AHR dimerizes with RelB in the production of IL-8. AHR or PPAR-γ activation promotes ubiquitination and degradation of p65/RelA. (3) Estrogen activates estrogen receptors (ER-α/β) to produce the NF-κB inhibitor, IκBα, and promotes the recruitment of ER-β to p65 binding sites, which blocks p65 transcriptional activity. (4) Heat shock protein (HSP)-90 is a chaperone shared by ER-α/β, PPAR-γ, AHR, and canonical NF-κB. (5) Elevated levels of p65/RelA, RelB, and HSP90 are found in CKD patient serum.

AHR interacts with both the canonical and non-canonical NF-κB pathways. The NF-κB transcription factor family consists of five proteins: p65 (RelA), RelB, c-Rel, p105/p50 (NF-κB1), and p100/p52 (NF-κB2). NF-κB1 and NF-κB2 are produced as inactive precursors, p105 and p100, respectively. RelB additionally associates with p100. Cell signals induce proteolytic processing of the inactive precursors, generating functional subunits p50 and p52. The Rel proteins, p65 and c-Rel, are similarly bound to inhibitory protein kinases (*e.g.,* IκBα, IκBβ and IκBε). Cell signal-induced phosphorylation of inhibitory kinases targets the kinases for degradation and release p65 or c-Rel. The various NF-κB proteins form transcriptionally active homo- and heterodimeric complexes. The p65 transcription factor most commonly associates with p50 but can also form homodimers or associate with c-Rel and p52. RelB is only known to form heterodimers with either p50 or p52. The canonical pathway involves the activation of p65/p50 whereas the non-canonical pathway involves RelB (Hayden and Ghosh, 2004; Oeckinghaus and Ghosh, 2009). In mouse embryonic fibroblasts, p65 and RelB exhibit negative crosstalk (Marienfeld et al., 2003; Jacque et al., 2005) and in mouse kidney fibroblasts, RelB suppresses the production of TNF (Xia et al., 1999), which is a common mediator in CKD (Stenvinkel et al., 2021) (Figure 5).

In the canonical NF-κB pathway, dioxin-activated AHR promotes p65 ubiquitination for degradation by either the proteasome or lysosome in mouse peritoneal macrophages, (Dominguez-Acosta et al., 2018). AHR also promotes the degradation of ER-α (Luecke-Johansson et al., 2017) and PPAR-γ (Dou et al., 2019), which are both involved in p65 inhibition. Specifically, PPAR-γ promotes p65 ubiquitination for targeted proteasome degradation (Hou et al., 2012) whereas estrogen induces the production of the NF-κB inhibitor, IκBα, and promotes the recruitment of ER-β to p65 binding sites, which blocks p65 transcriptional activity (Xing et al., 2012) (Figure 5). Because animal models of glomerulosclerosis treated with 17β-estradiol (Maric et al., 2004) or pioglitazone (Nemeth et al., 2019) exhibit reduced tubulointerstitial fibrosis, estrogen and PPAR-γ cell signals may regulate CKD pathogenesis by inhibiting canonical NF-κB signaling.

A common factor in ER-α/β (Powell et al., 2010), PPAR-γ (Nguyen et al., 2013) and AHR (Soshilov and Denison, 2011) cell signaling is heat shock protein (HSP)-90. This chaperone contributes to the stabilization of IκB kinase (IKK), which is needed for the dissociation of IκB from NF-κB (O’Neill et al., 2015). In chronic glomerulonephritis patients, serum levels of HSP90 and NF-κB are higher than those in healthy individuals. (Chebotareva et al., 2020). The HSP90 inhibitor, geldanamycin, significantly suppresses angiotensin II-induced p65 nuclear translocation in cardiac cells (Lee et al., 2010) and in a murine renal ischemia-reperfusion injury model, pre-treatment with the HSP90 inhibitor, AT13387, improved renal function compared to controls (O'Neill et al., 2015). Understanding the potential competition for HSP90 in the ER-α/β, PPAR-γ, NF-κB, and AHR pathways may help in identifying novel therapeutics for CKD (Figure 5).

In the non-canonical NF-κB pathway, dioxin-activated AHR can dimerize with RelB and promote IL-8 production in U937 macrophages. RelB:AHR complexes bind RelB:p52 response elements as well as AHR response elements (Vogel et al., 2007), highlighting the complexity of AHR in the non-canonical pathway. RelB is expressed in renal tubular epithelial cells and levels gradually increase with progressive fibrosis in a mouse renal fibrosis model with unilateral ureteral obstruction. RelB immunohistochemical staining in renal tubular epithelial cells was also positively correlated with the intensity of kidney fibrosis in biopsy specimens in a study of 34 CKD patients. Interestingly, the serum RelB levels from CKD patients also correlated with the intensity of renal fibrosis compared healthy controls (Sun D. et al., 2021). This dysregulated RelB response may also reflect changes in AHR cell signals that regulate fibrosis. Further investigation of RelB:AHR initiated cell signals, particularly in response to uremic AHR ligands, may offer insight into CKD pathogenesis.

## Conclusion

AHR is a pleotropic cell signaling molecule with diverse ligand-specific functions. The elevated levels of uremic solutes that act as AHR ligands during the progression of CKD highlight the significance of AHR activity in these diseases. AHR contributes to the biotransformation of molecules in the clearance of xenobiotics, and these processes deserve further exploration in kidney pathophysiology. AHR competition with HIF-1α for binding to ARNT in pro-inflammatory and anaerobic responses, together with AHR antagonism of TGF-β1 cell signaling in fibrogenesis, support potential targeting of AHR to slow CKD progression. In the NAD *de novo* biosynthesis pathway, AHR stimulates RNA expression and protein production of the rate-limiting enzyme, IDO1, and binds molecules along this pathway that accumulate in CKD. NAD is required for the activity of TIPARP, which is involved in the recruitment of an E3 ligase for ubiquitin-mediated proteasome degradation of AHR. It is possible that the extracellular release of kynurenine and NAMPT, rather than intracellular metabolic catabolism of these molecules into NAD, alters the functions of AHR in CKD. Further investigation of AHR regulation of ACE2 and the downstream effects of the ACE2 cleavage product, Ang-(1–7), on Mas and AT_2_R may identify novel therapeutic targets in hypertension and fibrosis. Because each of these AHR functions may be regulated by crosstalk with ER-α, PPAR-γ, or NF-κB subunits, a more refined assessment of the binding affinity and availability of HSP90 in these cell signaling pathways during CKD is warranted.
